# Medical students’ change in learning styles during the course of the undergraduate program: from ‘thinking and watching’ to ‘thinking and doing’

**Published:** 2012-09-30

**Authors:** Marcela Bitran, Denisse Zúñiga, Nuria Pedrals, Oslando Padilla, Beltrán Mena

**Affiliations:** 1 Centro de Educación Médica, Facultad de Medicina, Pontificia Universidad Católica de Chile (PUC); 2 Departamento de Recursos Humanos, PUC; 3 Departamento de Salud Pública, Facultad de Medicina, PUC

## Abstract

**Background:**

Most students admitted to medical school are abstract-passive learners. However, as they progress through the program, active learning and concrete interpersonal interactions become crucial for the acquisition of professional competencies. The purpose of this study was to determine if and how medical students’ learning styles change during the course of their undergraduate program.

**Methods:**

All students admitted to the Pontificia Universidad Católica de Chile (PUC) medical school between 2000 and 2011 (*n* = 1,290) took the Kolb’s Learning Style Inventory at school entrance. Two years later 627 students took it again, and in the seventh and last year of the program 104 students took it for a third time. The distribution of styles at years 1, 3 and 7, and the mobility of students between styles were analyzed with Bayesian models.

**Results:**

Most freshmen (54%) were classified as *assimilators* (abstract-passive learners); *convergers* (abstract-active) followed with 26%, whereas *divergers* (concrete-passive) and *accommodators* (concrete-active) accounted for 11% and 9%, respectively. By year 3, the styles’ distribution remained unchanged but in year 7 *convergers* outnumbered *assimilators* (49% vs. 33%). In general, there were no gender-related differences.

**Discussion:**

Medical students change their preferred way of learning: they evolve from an abstract-reflexive style to an abstract-active one. This change might represent an adaptation to the curriculum, which evolves from a lecture-based teacher-centered to a problem-based student–centered model.

## Introduction

Students’ approach to learning has been a topic of interest for medical educators for many decades.^1^–^7^ This is not surprising since the ability to learn is indispensable to becoming a doctor. During the undergraduate years, medical students have to become flexible life-long learners, able to gather and organize information from many sources and prepared to apply the relevant knowledge to the solution of the patients’ problems in a humanitarian healthcare context.

One of the most influential theories in the study of professional learning is the *Experiential Learning Theory* (ELT) proposed by David Kolb.^8^ According to ELT, learning is a process in which knowledge is created as a result of a recursive transformation of experience: concrete sensory experiences are the source of thinking and reflections; these ideas are organized and condensed into concepts and models from which new ideas for action originate. In this cycle of experiential learning, students ‘touch all bases’: they *experience*, *reflect*, *conceptualize* and *act* in a recursive process, which is sensitive to the learning context.^9^ Thus, rather than being confined to the cognitive realm, learning is viewed as an adaptive process between the individual and his environment that involves the entire person: thoughts, feelings, perceptions and behaviour.

In the dyadic model proposed by ELT, learning always entails a tension between two opposite states: *thinking* or *feeling* on the one hand, and *watching* or *doing* on the other. According to Kolb, the natural inclination of people for one of the two poles of each dyad defines their preferred way of learning, or learning style.^8^

People who are naturally inclined toward *thinking* and *watching* while learning are classified as *assimilators*. Those who also prefer *thinking* to *feeling* but, unlike *assimilators, are active* problem solvers (*‘thinking and doing’*) are c*onvergers. Divergers* (*‘feeling and watching’*) are reflective learners who pay more attention to relational than rational issues, and *accommodators* prefer *‘feeling’* and *‘doing’*. Although everybody uses all learning modes (*thinking, feeling, watching, and doing*) depending on the circumstances, the relative ease and interest for a given learning challenge or discipline will differ according to the predominant learning mode involved.^8^,^10^

The ability to ‘visit’ all learning modes is particularly valuable for medical students. Since medicine is a science-based profession oriented to the healthcare of persons, students have to master the basic sciences - for which abstract conceptualization and reflection are required - as well as the interpersonal relationships that entail the ability to connect empathetically with the patient and act effectively and efficiently in critical situations.

In the last two decades, many studies have been published on learning styles (LS) of medical students.^5^,^11^ However, their relevance to medical education has often been questioned.^12^–^14^ The skepticism arises in part from the fact that most studies address highly specific issues and lack an overall or systematic perspective. For example, many reports describe the LS of groups of medical students at some point in their study program,^15^–^24^ whereas others examine the relationship between LS and academic performance in specific subject matter.^25^–^28^

In the vast majority of studies, the learning style is considered a fixed characteristic of the student; therefore, issues such as the evolution of LS during the course of the study program or the influence of contextual and social variables on the way students learn have not been addressed.

The maturation of medical students as a learners is a crucial process, which is not well understood yet.^29^,^30^ It is particularly relevant for some European and Latin American countries like Chile, where students enter medical school right after high school and remain in the program for 6 to 7 years. In Pontificia Universidad Católica de Chile (PUC) the seven-year undergraduate medical curriculum consists of two and half years of basic science courses, followed by another two and half years of preclinical courses and supervised clinical practice. Years 6 and 7 correspond to the internship, a supervised full clinical practice.^31^ Starting in year 3 a new learning paradigm is introduced that incorporates most of the educational strategies of the SPICES model.^32^ The lecture-based instruction is gradually replaced by experiential learning, which involves students’ interaction with several people (from faculty to patients) in different settings (small group seminars, hospital and ambulatory settings) and evaluation of knowledge, practical and relational skills, and attitudes.^31^ The ability to adapt to this change is crucial and the costs and benefits involved are likely related to the students’ learning styles. This task might be particularly challenging for students who are not naturally prone to using *concrete* and *active* learning modes, like the majority of those accepted to medical school.^33^

The implication of this kind of study is not for faculty members to change their teaching style, but for students to understand their own mental process in relation to the changing learning scenarios. The job of teachers is to help students become independent learners, critical thinkers, who understand their own mental processes and the thought process of others.

The present study was aimed at answering the question of whether medical students change their learning style preference during the course of the study program. Here, we report the results of both a cross-sectional study that compared the LS of students in years 1, 3 and 7 of the study program, and the follow-up of 104 students at a later stage of the program. The stability of LS over time at a population level (i.e., the general distribution of LS) and at an individual level, were analyzed, as well as the influence of gender.

## Methods

### Participants

All students admitted to medical school from years 2000 to 2011 (*n* = 1,290, 41% females, 59% males; age at admission: 18.6 (*SD* 1.0 years). According to their scores on the Prueba de Selección Universitaria, (the national university admission test), these students belong in the upper 1% of Chilean students in terms of academic performance.^33^

### Instrument

Learning styles were determined with the Learning Style Inventory (LSI, Spanish version).^10^ All students admitted to the PUC medical school between 2000 and 2011 (*n* = 1,290) took the Kolb’s Learning Style Inventory at school entrance. Of these, 627 were retested two years later and 104 were tested for a third time in the seventh and last year of the program. This group of 104 students belonged to the cohorts admitted in 2000–2001. At each testing occasion, all participants gave their informed consent. A medical educator administered the tests during teaching hours and students received a report with their results.

### Data analysis and statistics

The data were analyzed with Bayesian statistics using WINGBUGS. We selected this type of analysis because the data included events whose frequency was null. In such cases, confidence intervals could not be obtained with classical statistics. We calculated the probability and 95% credibility intervals of each learning style in years 1, 3 and 7 of the study program, as well as the probability of students moving from one style to another. The gender variable was included in the model to allow for comparisons between male and female students. To compare results from two given conditions, the credibility interval for the differences between probabilities was calculated. A difference between two probability values was considered statistically significant if the corresponding credibility interval did not include the zero value.

## Results

### Students’ learning styles distribution over the course of the undergraduate study program

**Figure 1:**
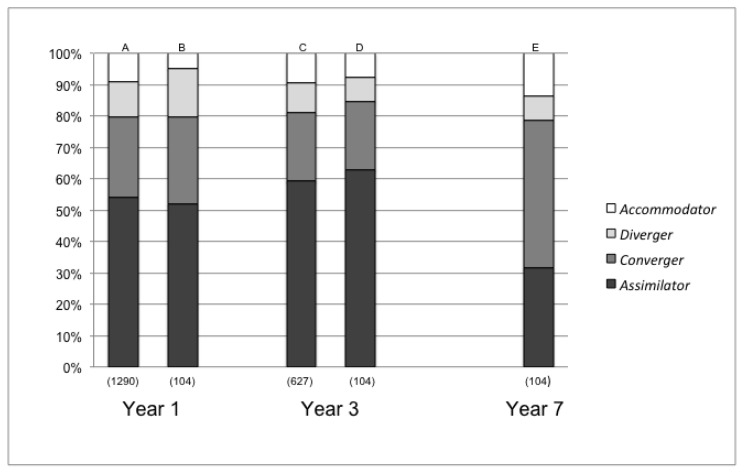
Distribution of learning styles at years 1, 3 and 7 of the program Notes: a. The learning styles’ distribution at admission of the 1,290 students matriculated between 2000 and 2011; b: Learning styles’ distribution at admission of 104 students followed throughout the study program; c: Learning styles’ distribution at year 3 of 627 students tested at admission; d: Learning styles’ distribution at year 3 of 104 students followed throughout the study program; e: Learning styles’ distribution at year 7 of the 104 students followed throughout the study program. The number of students is indicated in parenthesis.

More than half of the 1,290 students who entered the PUC medical school between 2000 and 2011 classified as *assimilators* at admission (Figure 1, column A). The second most frequent was *convergers* (26%); *divergers* accounted for 11%, and *accommodators* formed the smallest number (9%) (Fig 1, column A).

The twelve cohorts studied between 2000 and 2011 displayed a similar distribution of learning styles at admission (only 24 out of 254 possible comparisons tested significantly; data not shown).

The distribution of learning styles of third-year students *(n* = 627) was similar to that of freshmen, with the *assimilators* being the most numerous (59%; Figure 1, column C). However, at the end of the study program (Figure 1, column E) the largest number were *convergers* (increasing from 26% in year 1 to 49% in year 7) at the expense of the number of *assimilators* who ranked second (from 54% in year 1 to 32% in year 7).

Identical results were found with the group of 104 students who took the test on all three occasions (Figure 1, columns B, D and E). At admission, the *assimilating* style prevailed over the other three styles. The pattern remained the same by year 3 but changed in year 7, when *convergers* outnumbered *assimilators*.

### Learning styles distribution by gender over the course of the study program

The distribution of learning styles of medical students in years 1, 3 and 7 grouped by gender is reported in Table 1. There were no differences in the proportions of female and male student for any style at any of the times studied. On the other hand, the number of both male and female *assimilators* decreased from year 1 to year 7 (from 46% to 30% in female and from 58% to 33% in male students), while a concomitant increase in the proportion of *converging* male learners occurred (from 21% to 47%; see Table 1). Female students displayed a similar trend.

### Mobility of medical students between learning styles

The estimated mobility of students between the four learning styles from year 1 to year 3 of the study program according to gender is shown in Table 2. The *assimilating* style was the most stable of all styles; more than 70% of students who classified as *assimilators* at school entrance kept their preference until year 3, whereas only 46% of male and 34% of female students who were *convergers* at admission retested as such two years later. On the other hand, fewer than 25% of *accommodators* and *divergers* kept their style. Independent of their preference at school entrance, most of the students who changed styles became *assimilators* by year 3 (Table 2).

The general pattern of mobility of male and female students from year 1 to year 3 was similar with few exceptions. A larger proportion of female than male *accommodators* became *convergers* (29% vs. 2%%, *p* < 0.05); more male than female *convergers* became *divergers* (15% vs. 1%%, *p* < 0.05) and more male than female *divergers* became *assimilators* (56% vs. 33%, *p* < 0.05) (Table 2).

The estimated mobility of students between learning styles from year 1 to year 7 of the study program according to gender is displayed in Table 3. Over these six years, the *converging* style was the most stable of all four styles; 54% of female and 65% of male students initially classified as *convergers* kept their preference from admission until year 7. The retention of *assimilators* was 40% and 45% for both female and male students, respectively. Interestingly, none of the *accommodators* retained their style. Most of the students who changed styles between admission and year 7 became *convergers* (Table 3). There was only one gender-related difference in terms of mobility during this time interval: whereas 6% of male *assimilators* became *divergers*, none of the female *assimilators* did (see Table 3).

### Generalizability

With regard to their learning styles distribution at admission, the 627 students retested at year 3 were indistinguishable from the 633 individuals who did not partake in this second measurement. In addition, there were no differences between these two groups in terms of gender composition. Similarly, no differences were detected in terms of learning styles between the students who took the test on all three testing occasions (*n* = 104) and those who did not answer the questionnaire in the last test application (*n* = 1,182). In terms of gender composition, there was a slight overrepresentation of female students in the group of 104 students followed throughout the program (11% of male students vs. 7% of female students).

## Discussion

Learning styles (LS) of medical students changed markedly over the course of the undergraduate study program: from a majority of *assimilators* (abstract-passive learners) at admission, to a preponderance of *convergers* (abstract-active learners), by the end of the program. This was evidenced by the cross-sectional comparison of LS of 1^st^, 3^rd^ and 7^th^-year undergraduates and also by the longitudinal follow-up of 104 of these students at a later stage of the study program. This activation occurred somewhere between years 3 and 7, a period characterized by the progressive incorporation of teaching and evaluation methodologies that require active student participation and development of interpersonal skills.^31^

The transition analyses revealed that most of the increase in the number of *convergers* was explained by a massive transformation of students who tested as *assimilators* at admission. We propose that this transformation responds to the increasing demand for active learning and engagement that characterizes the later years of the curriculum, in preparation for the upcoming professional challenges. This idea is consistent with the report by Engels *et al.*^24^ of their study at a Canadian medical school, which indicates that the *converging* style – more oriented to problem solving - prevailed among surgery residents and faculty, whereas most medical students were *assimilators*. This preference for active learning activities has also been reported for residents of other medical specialties, such as dermatology.^20^ In addition, present results are consistent with a cross-sectional study done in Iran showing that final-year medical students have a larger proportion of active learners than freshmen.^22^ In contrast, a longitudinal study of Irish pre-registration nursing students showed that most of them maintained the ‘reflector’ learning style from first until the fourth and final year.^34^ The reasons for this difference are not known; however, they may relate to the differences in the instruments used to identify LS and/or differences associated with the students and/or the curriculum.

Furthermore, an activating effect similar to the one reported here was shown a long time ago in undergraduate students of arts, who became participative learners by the end of the program.^35^ Thus, the changes seen in medical students may be an example of a general phenomenon, common to professional careers and related to the curricular modifications purposely implemented toward the end of the program, to foster the development of professional skills.

The idea that the curriculum can influence the learning patterns and preferences of students is supported by a longitudinal study showing that the introduction of an integrated medical curriculum was associated with an increase in self-regulation strategies and vocational orientation of medical students.^23^

It can be argued that rather than adaptation of the learner to the curriculum, the changes of LS observed between admission and graduation could relate to other factors such as poor reliability of the instruments^13^ or age-dependent maturation of students independent of curricular influences. The first explanation seems unlikely. Results from this and other studies indicate that Kolb’s LS scores are stable for test-retest intervals lasting from ten weeks to a year.^36^ Furthermore, the remarkable stability of the pattern of LS of entering student cohorts seen for the last twelve years in the PUC medical school, argues strongly in favor of a good reproducibility of Kolb’s LS inventory.

Evidently, the effects of maturation and adaptation to curricular changes are difficult to isolate from each other. On the one hand, few longitudinal studies are available for comparison purposes. On the other hand, from a technical point of view, it is difficult to design a study with proper control groups exposed to medical curricula that do not evolve to include more active learning tasks. We think that maturation and adaptation to learning requirements contribute interactively to the changes observed in this study and others, in a way that is consistent with the concept of ‘learning spaces’ proposed in 2005 by David Kolb. He suggested that rather than ‘having’ a given and fixed style, learners ‘move’ across a space of possibilities.^9^,^37^ The style people build early on in life would correspond to their ‘home base’ and, as they mature and face new challenges, they will start to ‘visit’ non-preferred sites of the learning space.

The adaptation thesis can also explain the stability of *assimilators* during the first 2 years of medical studies unveiled here by the mobility analysis. This period is characterized by lecture-based teaching and memory-based evaluations,^31^ for which *assimilating* learners are well prepared 8 Consequently, these learners would have no reason to visit other ‘places’ and change a style that gives them an advantage for academic performance. The situation of concrete and active students during this period is different: to learn basic sciences they must use their less preferred skills: abstract conceptualization and reflexive observation. Thus, to meet this challenge many of them migrated to the abstract-reflexive corner, home to the *assimilator*. In fact, more than half of *accommodators* (concrete-active learners) and 40 % of *divergers* (concrete-reflective learners) become *assimilator*s by year 3. The adaptive value of using an *assimilating* style during the first two years of study is consistent with the observation that these learners perform best in multiple choices tests,^27^ the evaluation instrument most often used in these initial years.

Students who ‘visit’ different styles when faced with new learning challenges add new learning skills to their repertoire. In the case reported here, students who shifted from *assimilators* to *convergers* would be better prepared to ‘learn by doing’ while being ready to use their trusted reasoning skills when required.

The main merit of this study is the inclusion of a transition analysis that makes visible the dynamic nature of the changes. Most of the published studies on LS report only the distributions of students at different times. This static view does not tap into the issue of the turnover of students between styles. This is of the utmost importance because a similar LS distribution at different testing times might be erroneously interpreted as indicative of no change. This similarity could also result from the reciprocal exchange of an equal number of students between styles.

Other positive aspects of this study are the inclusion of both a cross-sectional and a longitudinal study, and analysis of generalizability that allows us to say that at each time studied the students who answered the tests were indistinguishable from those who did not in terms of LS and gender, thus validating the applicability of present findings to all the students of PUC medical school.

We are aware of some of the limitations of the present study. First, some readers may not be acquainted with the statistical analysis used in this study. As explained in the Methods section, classical statistics could not be used because of the nature of the results. For instance, using Bayesian statistics, we calculated that no more than 4% of female assimilators in year 1 became diverging learners in year 7 (Table 3). This information could not have been obtained with classical statistics. Second, caution is needed when directly comparing the results reported here with other studies because there are differences in duration of medical studies between Chile and other countries. However, in terms of age and clinical experience, it is safe to say that seventh-year medical students from Chile correspond to fourth year North American medical students and 5th year medical students of some European countries. Third, this study was based on one medical program; therefore, it should be replicated in several institutions before its generalizability can be established. In addition, there may be cultural influences on LS of students and their transformation, which may affect the global value of the findings reported here.

To our knowledge, this is the first complete longitudinal follow-up study of medical students’ LS reported in Latin America. We think that the present findings will likely be applicable to other universities in Chile and the region, given the similarity of medical students’ profiles at admission in terms of learning styles and age.^38^–^41^

### Conclusion

Most medical educators and faculties would agree that helping students become flexible life-long learners is an important responsibility of medical schools. Students need to relate to all forms of knowledge employing different learning modes as needed. The progression through the curriculum stresses the importance of ‘learning by doing’ (or using *active experimentation*) and some educators would go even further to say that clinical tutors’ behaviour is key to fostering student’s openness to criticism and experimentation.^42^ Helping *abstract* learners to do this might be particularly advisable because abstract learners have been reported to be less flexible than *concrete* learners.^9^ Thus, using Experiential Learning Theory as a framework, many interventions can be designed to foster integrated learning, a process where learners “touch all the bases” - experiencing, reflecting, thinking, and acting - in a recursive process that is sensitive to the learning situation.^9^,^37^

We hope this research contributes to enhanced awareness of medical educators about the tensions that medical students experience to respond to the changing learning demands across the curriculum. The results reported here confirm the idea that the challenge is not the same for all students at any given time, nor for the same student at different times. It depends amongst other factors on their learning home base and their flexibility to ‘march to a different drummer’, according to the demands of the learning task at hand.

The implications of these results are not for medical educators to change their teaching style to suit the modifications in their students’ learning styles. Rather, it is to understand that a medical student is a complex and dynamic ‘system’. The challenges for teachers are 1) to realize that a well-designed instruction addresses various learning preferences to promote individual learning throughout a group of learners and 2) to be aware of the tensions facing students on their way to become life-long learners and acquire the professional competencies of a doctor.

## Figures and Tables

**Table 1:** Distribution of Learning styles among female and male students at years 1, 3, and 7

Learning Styles	Students’ Sex	Year 1 *(n* = 1,290)	Year 3 *(n* = 627)	Year 7 *(n* = 104)
**Accommodator**	female	9% (6 – 13)	10% (6 – 13)	18% (9 – 28)
	male	8% (6 – 11)	9% (7 – 12)	11% (4 – 20)

**Assimilator**	female	46% (41 – 52)	53% (47 – 59)	30% (19 – 42)
	male	58% (53 – 63)	63% (58 – 68)	33% (21 – 46)

**Converger**	female	29% (24 – 35)	28% (23 – 33)	44% (31 – 57)
	male	21% (17 – 26)	17% (14 – 22)	47%** (34 – 60)

**Diverger**	female	15% (11 – 19)	9%* (6 – 13)	9% (3 – 17)
	male	12% (9 – 16)	10% (7 – 13)	9% (3 – 18)

* *P* < 0.05 relative to Year 1

** *p* < 0.05 relative to Year 1 and 3

In parentheses is the 95% credibility interval

**Table 2:** Matrix of mobility between learning styles among female and male students from year 1 to year 3

Learning Styles Year 1	Students’ Sex	Learning Styles Year 3			

		Accommodator	Assimilator	Converger	Diverger
**Accommodator**	female	15% (5 – 33)	46% (27 – 65)	29%* (13 – 49)	7% (1 – 22)
	male	20% (8 – 37)	66% (47 – 81)	2% (0 – 12)	9% (2 – 24)

**Assimilator**	female	3% (1 – 7)	70% (62 – 78)	17% (11 – 24)	10% (5 – 15)
	male	6% (3 – 10)	73% (67 – 79)	14% (9 – 18)	7% (4 – 11)

**Converger**	female	12% (6 – 21)	40% (29 – 51)	46% (35 – 57)	1%* (0 – 5)
	male	10% (5 – 18)	40% (30 – 51)	34% (24 – 45)	15% (8 – 24)

**Diverger**	female	18% (8 – 31)	33% (20 – 48)	25% (13 – 40)	23% (12 – 37)
	male	13% (6 – 26)	56% (41 – 70)	16% (7 – 29)	14% (5 – 25)

* *P* < 0.05 relative to male students

In parentheses is the 95% credibility interval

**Table 3:** Matrix of mobility between learning styles among female and male students from year 1 to year 7

Learning Styles Year 1	Students’ Sex	Learning Styles Year 7			

		Accommodator	Assimilator	Converger	Diverger
**Accommodator**	female	0% (0 – 100)	0% (0 – 25)	100% (66 – 100)	0% (0 – 10)
	male	0% (0 – 18)	45% (2 – 100)	51% (3 – 98)	0% (0 – 20)

**Assimilator**	female	18% (7 – 35)	40% (24 – 60)	41% (23 – 59)	0%* (0 – 4)
	male	6% (0 – 20)	45% (27 – 63)	40% (24 – 59)	6% (1 – 19)

**Converger**	female	12% (2 – 33)	26% (9 – 51)	54% (29 – 77)	4% (0 – 22)
	male	5% (0 – 24)	27% (9 – 53)	65% (39 – 86)	0% (0 – 5)

**Diverger**	female	23% (4 – 59)	9% (0 – 41)	23% (4 – 57)	37% (10 – 70)
	male	22% (4 – 57)	0% (0 – 9)	50% (18 – 82)	23% (4 – 58)

* *P* < 0.05 relative to male students

In parentheses is the 95% credibility interval
